# Supplementary material for: Region-specific regulation of stem cell-driven regeneration in tapeworms
Source: eLife. 2019 Sep 24;8:e48958. doi: 10.7554/eLife.48958 (PMC6821492; doi:10.7554/eLife.48958)
Supplement: Reporting standard 1. [file elife-48958-repstand1.pdf]

MINSEQE: Minimum Information about a high-throughput Nucleotide SeQuencing Experiment - a proposal for standards in functional genomic data reporting

Version 1.0 (June 2012)

Nucleotide sequencing is now a mainstay technology in modern biology. In the past several years, the cost of High-Throughput Sequencing (HTS) has steadily decreased and the technology has gained tremendous popularity. HTS is now being used not only for traditional applications in genomics, but also to assay gene expression (RNA-Seq), transcription factor binding, DNA methylation or chromatin modifications (ChIP-Seq), and will soon be employed for clinical purposes such as diagnostics. As the cost of instrumentation and reagents continues to decrease and the technology becomes accessible to individual laboratories, we anticipate an even more dramatic rise in the use of HTS for a variety of assays.

HTS based assays generate vast quantities of digital data, the interpretation of which requires a solid understanding of the biological samples used, as well as of the analyses carried out on the data. Availability of these data in usable formats through public archives such as ArrayExpress or Gene Expression Omnibus is essential not only for proper peer-review of the data and to ensure experimental reproducibility, but to allow integration of multiple experiments across multiple modalities, therefore maximising the value of high-throughput research. In particular, investigators frequently access these databases to ask questions of the data that were not anticipated by the original depositor or that can only be asked by querying across multiple data sets. This can only be achieved by adopting reporting guidelines for describing, storing, and exchanging high-throughput data.

The availability and usability of microarray data was greatly facilitated by the adoption of the Minimum Information About a Microarray Experiment (MIAME) guidelines by leading scientific journals. However no similar guidelines have been so far adopted for HTS applications. Here we propose guidelines for the minimum information that should be reported about HTS experiments ("MINSEQE") to enable their unambiguous interpretation. Since describing such experiments is not fundamentally different from analogous microarray experiments, we can benefit from the well-developed and broadly accepted MIAME guidelines [1,2]. We propose that the following five elements of experimental description are considered essential for making available data supporting HTS based publications.

1. The description of the biological system, samples, and the experimental variables being studied.

Essential sample annotation, including the *experimental factors* and their values, must be given. Experimental factors are the key experimental variables, e.g. "compound" and "dose" in dose-response experiments or "antibody" in ChIP-Seq experiments. In addition to experimental factor values, essential information about the biological system from which samples were taken must be given, e.g. the organism, strain or cultivar (if known and if appropriate), the organism part or tissue, and what treatment(s) was/were applied.

2. The sequence read data for each assay.

This represents the complete set of read sequences and base-level quality scores for each assay, as generated by the sequencing instrument, in a recognized format. Currently FASTQ format is recommended, with a description of the scale used for quality scores.

3. The 'final' processed (or summary) data for the set of assays in the study.

The final processed data is defined as the data on which the conclusions in the related publication are based. Currently there are no widely adopted formats for processed HTS data. Until such standardized formats become available, it is essential that descriptions of the data format be provided. For gene expression, in many cases these data can be presented as a matrix with each row corresponding to a genomic region (such as a gene or exon), each column representing a particular biological state (e.g. a time point in a time course experiment), and each element in the matrix representing a measurement of the particular genomic region in the particular biological state. Similarly, other applications like ChIP-Seq analyses typically generate tabular output for identified peaks mapped to a reference sequence.

4. General information about the experiment and sample-data relationships

General information about the overall study includes a summary of the experiment and its goals, contact information, and any associated publication. Part of this description should be a table specifying sample-data relationships, i.e. which sample has led to which raw data file or which data element in the processed data files.

5. Essential experimental and data processing protocols

Experimental processing methods should describe how the nucleic acid samples were isolated, purified and processed prior to sequencing. As each of the different sequencing platforms use technologies that are based on highly divergent chemistries, it is recommended that a summary of the instrumentation used, library preparation strategy, labelling and amplification methodologies, alignment algorithms and data filtering used be provided. Data processing and analysis protocols must be described in sufficient detail to enable unambiguous data interpretation and to enable scientists to reproduce the analysis steps. This should include, but is not limited to, data rejection methods, data correction methods, alignment methods, data smoothing and filtering methods and identifiers used for reference genomes to which the sequences were mapped (when applicable).

These core descriptions should be required by journals and checked by editors and reviewers, by archives and checked by curators or scripts, and by data-generating consortiums and checked by data coordinating centers. Templates and checklists should be made available and shared to facilitate the process. The Functional Genomics Data (FGED) Society can aid in this effort.

In conclusion, the adoption of a standard such as we recommend above will be essential for biological research using HTS in coming years, especially to enable data integration. Corresponding to usage of MIAME in microarray experiments and the public archives, widespread adoption of MINSEQE will greatly aid integration of HTS experiments into repositories for data mining, transforming how HTS results can be queried.

References.

[1] Brazma et al, Minimum information about a microarray experiment (MIAME)—toward standards for microarray data, Nature Genetics, 29, 365 - 371 (2001).

[2] http://fged.org/projects/miame/
