# Supplementary material for: Region-specific regulation of stem cell-driven regeneration in tapeworms
Source: eLife. 2019 Sep 24;8:e48958. doi: 10.7554/eLife.48958 (PMC6821492; doi:10.7554/eLife.48958)
Supplement: Transparent reporting form [file elife-48958-transrepform.docx]

***eLife’s* transparent reporting form**

We encourage authors to provide detailed information *within their submission* to facilitate the interpretation and replication of experiments. Authors can upload supporting documentation to indicate the use of appropriate reporting guidelines for health-related research (see [EQUATOR Network](http://www.equator-network.org/%20)), life science research (see the [BioSharing Information Resource](https://biosharing.org/)), or the [ARRIVE guidelines](http://www.plosbiology.org/article/info:doi/10.1371/journal.pbio.1000412) for reporting work involving animal research. Where applicable, authors should refer to any relevant reporting standards documents in this form.

If you have any questions, please consult our Journal Policies and/or contact us: [editorial@elifesciences.org](mailto:editorial@elifesciences.org).

**Sample-size estimation**

- You should state whether an appropriate sample size was computed when the study was being designed
- You should state the statistical method of sample size computation and any required assumptions
- If no explicit power analysis was used, you should describe how you decided what sample (replicate) size (number) to use

Please outline where this information can be found within the submission (e.g., sections or figure legends), or explain why this information doesn’t apply to your submission:

No explicit power analysis was used to determine sample sizes. Sample sizes were deemed adequate as the differences between control and experimental groups were significant and reproducible.

**Replicates**

- You should report how often each experiment was performed
- You should include a definition of biological versus technical replication
- The data obtained should be provided and sufficient information should be provided to indicate the number of independent biological and/or technical replicates
- If you encountered any outliers, you should describe how these were handled
- Criteria for exclusion/inclusion of data should be clearly stated
- High-throughput sequence data should be uploaded before submission, with a private link for reviewers provided (these are available from both GEO and ArrayExpress)

Please outline where this information can be found within the submission (e.g., sections or figure legends), or explain why this information doesn’t apply to your submission:

All experiments were repeated at least twice. Number of experiments (N) and number of samples (n) are indicated in the legends.

No data was excluded except for in Figure 4e: Some RNAi worms had degenerated necks and we did not include them for quantification of cycling cells. This is stated in the figure legend. Should we have included all the RNAi animals, the significance of the results would not have changed. However, we deemed it inappropriate to include these animals that had few or no cycling cells once the neck morphology was severely affected.

qPCR in Figure 4 – figure supplement 1 was performed with two biological replicates (of three pooled worms per RNAi condition) and three technical replicates per biological replicate.

**Statistical reporting**

- Statistical analysis methods should be described and justified
- Raw data should be presented in figures whenever informative to do so (typically when N per group is less than 10)
- For each experiment, you should identify the statistical tests used, exact values of N, definitions of center, methods of multiple test correction, and dispersion and precision measures (e.g., mean, median, SD, SEM, confidence intervals; and, for the major substantive results, a measure of effect size (e.g., Pearson's r, Cohen's d)
- Report exact p-values wherever possible alongside the summary statistics and 95% confidence intervals. These should be reported for all key questions and not only when the p-value is less than 0.05.

Please outline where this information can be found within the submission (e.g., sections or figure legends), or explain why this information doesn’t apply to your submission:

All replicates, samples sizes, error bars, statistical tests and p-values are described in the corresponding figure legends, with an additional section in the Materials and Methods. When two groups were compared, we used Student’s t-test. When three or more groups were compared, we used one-way ANOVA. ANOVAs were also performed with multiple comparison testing: Dunnett’s test was performed when comparing means of multiple samples to one control whereas Tukey’s test was performed when comparing all means to all other means, as recommended by the statistical software we used (Prism7).

(For large datasets, or papers with a very large number of statistical tests, you may upload a single table file with tests, Ns, etc., with reference to sections in the manuscript.)

**Group allocation**

- Indicate how samples were allocated into experimental groups (in the case of clinical studies, please specify allocation to treatment method); if randomization was used, please also state if restricted randomization was applied
- Indicate if masking was used during group allocation, data collection and/or data analysis

Please outline where this information can be found within the submission (e.g., sections or figure legends), or explain why this information doesn’t apply to your submission:

Not relevant

**Additional data files (“source data”)**

- We encourage you to upload relevant additional data files, such as numerical data that are represented as a graph in a figure, or as a summary table
- Where provided, these should be in the most useful format, and they can be uploaded as “Source data” files linked to a main figure or table
- Include model definition files including the full list of parameters used
- Include code used for data analysis (e.g., R, MatLab)
- Avoid stating that data files are “available upon request”

Please indicate the figures or tables for which source data files have been provided:

Source data not provided. We typically plotted raw data points in all graphs except for Fig 1c-d, where averages and standard deviations were plotted to make the graph more readable.
